# Risk Assessment of Venous Thromboembolism among Septic Shock Patients: Single versus Concurrent Insertion of Central Venous Catheters

**DOI:** 10.3390/medicina60050785

**Published:** 2024-05-09

**Authors:** Cosmin Iosif Trebuian, Adina Maria Marza, Alexandru Cristian Cindrea, Alina Petrica, Stefania Onea, Dumitru Sutoi, Claudiu Barsac, Iulia Crintea-Najette, Daian Popa, Raul Chioibas, Ovidiu Alexandru Mederle

**Affiliations:** 1 Department of Surgery, “Victor Babes” University of Medicine and Pharmacy, 300041 Timisoara, Romania; trebuian.cosmin@umft.ro (C.I.T.); alexandru.cindrea.umfvbt@gmail.com (A.C.C.); alina.petrica@umft.ro (A.P.); dumitru.sutoi@umft.ro (D.S.); claudiu.barsac@umft.ro (C.B.); iulia.crintea@umft.ro (I.C.-N.); daian-ionel.popa@umft.ro (D.P.); chioibas.raul@umft.ro (R.C.); mederle.ovidiu@umft.ro (O.A.M.); 2 Department of Anesthesia and Intensive Care, Emergency County Hospital, 320210 Resita, Romania; 3 Emergency Department, Emergency Clinical Municipal Hospital, 300079 Timisoara, Romania; 4 Emergency Department, “Pius Brinzeu” Emergency Clinical County Hospital, 300736 Timisoara, Romania; 5 Clinic of Anesthesia and Intensive Care, “Pius Brinzeu” Emergency Clinical County Hospital, 300736 Timisoara, Romania; stefania.onea@student.umft.ro

**Keywords:** venous thromboembolism, Caprini risk score, concurrent CVCs, septic shock, hemodialysis catheters

## Abstract

*Background and Objectives:* Thrombosis is a serious complication experienced by some hospitalized patients. While concurrent placement of two catheters (CVCs) in the same central vein offers several benefits in clinical settings, we aimed to investigate the role of this procedure in relation to the risk of thrombosis. *Materials and Methods:* Over a two-year retrospective analysis, we examined 114 patients with septic shock caused by a pulmonary infection, who underwent the insertion of one or more central lines into a central vein during their ICU stay. Logistic regression models were employed to assess the correlation between the Caprini risk score, the placement of two CVCs in the same vein, COVID-19 infection and the risk of venous thromboembolism (VTE). *Results:* In total, 53% of the patients underwent the concurrent insertion of two CVCs. The placement of two CVCs in the same vein appears to elevate the VTE risk by 2.5 times (95% CI: 1.03–6.12). Logistic regression analysis indicated that hemodialysis catheters amplify the VTE risk by nearly five times, even when accounting for a series of factors (95% CI: 1.86–12.31). *Conclusions:* Our study suggests that the elevated risk of VTE is likely associated with the insertion of the hemodialysis catheters rather than solely the presence of two concurrent catheters.

## 1. Introduction

Thrombosis is a major concern in hospitalized patients, especially in those experiencing septic shock. In certain situations, within the intensive care unit (ICU), the simultaneous placement of central venous catheters (CVCs) for drug and fluid infusion, along with central venous hemodialysis catheters (CV-HDCs) for kidney replacement therapy, may be used [1,2]. This possibility may raise concerns about an elevated risk of complications including thrombosis, infections, malposition, pneumothorax, vein stenosis, numerous punctures, or even CV-HDC dysfunction [3].

In the last few years, a significant group of patients who are admitted to the ICU and require CVC or CV-HDC placement are those with severe manifestations of COVID-19 disease, who experience respiratory failure, septic shock, multiple organ dysfunction [4]. The hypercoagulable state in patients with SARS-CoV-2 infection is a well-known risk factor for arterial and venous thromboembolism [5], especially in those with advanced vascular access devices [6].

The reported incidence of catheter-related venous thromboembolism (VTE) varies widely, from 9.5 per 1000 catheter days in critically ill patients [7] to 5–13.5% in oncology and hematological patients [8,9].

However, the thrombotic risk among individuals with one catheter versus those with two catheters in the same vein remains unclear [10], but their simultaneous placement could have some benefits. Notable among these considerations is minimizing the patient’s discomfort by preparing them only once for the procedure. This approach limits the duration of doctor–patient interaction, particularly important for patients with COVID-19 to decrease the risk of staff contamination. It is essential to promptly establish reliable vascular access and initiate hemodialysis simultaneously in some critical situations like septic shock while also prioritizing the preservation of other central veins, essential for the subsequent period of the ICU stay.

The Caprini risk score, first published in 1991, is a thrombotic risk assessment tool validated in over 250,000 surgical patients [11]. Its usefulness was proven in multiple clinical scenarios, including plastic surgery and perioperative care [12,13], but numerous studies utilized the Caprini score also in non-surgical patients to quantify the mortality risk in hospitalized patients [14], including in those with SARS-CoV-2 infection [15,16,17].

In a recent study conducted by Sebolt et al. [15], the Caprini risk score was employed to assess the risk of VTE in 1228 COVID-19 patients admitted to 40 different hospitals. Among these patients, 261 received at least one vascular access device. The study revealed that hospitalized patients with COVID-19, who received vascular access devices, had significantly higher rates of VTE compared to those who did not, with rates of 21.5% and 6.1%, respectively [15].

Our main objective was to analyze the risk of VTE in patients with septic shock caused by a pulmonary infection considering the Caprini risk score, the placement of two CVCs in the same vein and SARS-CoV-2 infection status. Our secondary objectives aimed to assess the ability of the Caprini risk score to predict in-hospital death or prolonged hospitalization.

## 2. Materials and Methods

### 2.1. Study Design and Patients

**Figure 1:**
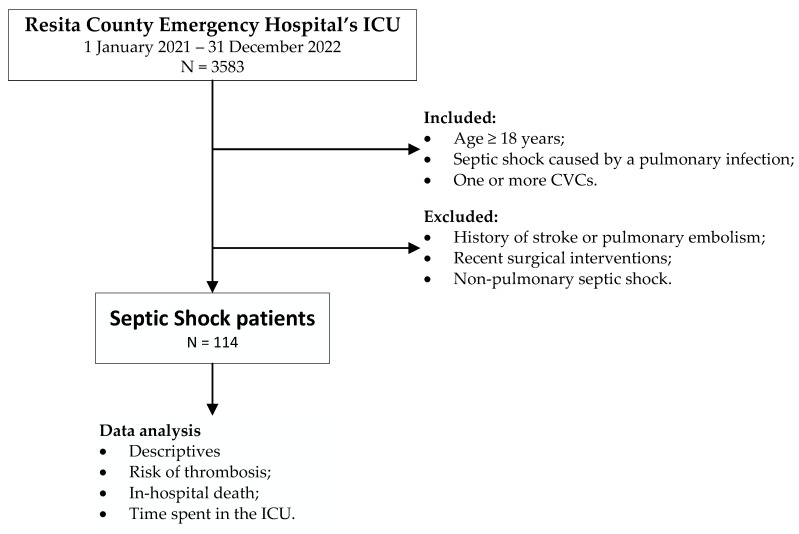
Study flow diagram. The following abbreviations are used: CVCs—central venous catheters, ICU—intensive care unit.

A retrospective analysis of the electronic medical records (EMRs) from 1 January 2021 to 31 December 2022 was performed at Resita County Emergency Hospital. A total of 3583 patients were admitted to the ICU during this period of time. Through a keyword-based search, 114 patients with pulmonary septic shock were initially identified and, subsequently, they underwent manual verification to ensure they met the inclusion and exclusion criteria. The selection was performed according to the following inclusion criteria: age equal to or above 18 years, septic shock caused by a pulmonary infection, one or more central vascular access routes. The exclusion criteria were age below 18 years, diagnosis of stroke and/or pulmonary embolism upon ICU admission, septic shock caused by other source but the lungs, recent surgical intervention (within 2 weeks). The study flow diagram is presented in Figure 1.

The diagnosis of septic shock was established based on the necessity for a vasopressor to maintain a mean arterial pressure (MAP) of ≥65 mmHg [18] and an elevated serum lactate level surpassing 2 mmol/L, in patients with confirmed or high suspicion of infection. Considering the study was conducted during the pandemic, an important proportion of the patients with septic shock admitted to the ICU had COVID-19, often exhibiting lung damage exceeding 50% of the lung’s surface. Consequently, we restricted the study to include only patients with septic shock originating from pulmonary infections.

VTE was primarily diagnosed through Doppler ultrasonography. In instances where pulmonary embolism was suspected due to suggestive symptoms, clinical signs or a sudden elevation in D-dimers, chest angio-CT along with echocardiography served as the primary diagnostic tools.

In certain situations, to facilitate therapeutic interventions such as hemodialysis or plasmapheresis, as well as the administration of fluid and vasopressors, a subgroup of patients required the insertion of two vascular accesses in the same central vein. Using ultrasound guidance, both midline catheters and hemodialysis catheters were placed as part of the same procedure in 60 patients, specifically in the right internal jugular vein or femoral vein. Seven French multi-lumens CVCs, the Certofix Protect Trio by Braun (Kronberg, Germany), were used for perfusion, and 12 French Haemocat Signo catheters were available for hemodialysis and plasmapheresis procedures. Plasmapheresis was usually recommended for patients diagnosed with autoimmune disorders and hematological diseases. Patients requiring kidney replacement therapy underwent continuous veno-venous hemodiafiltration (CVV-HDF) as part of their therapeutic approach.

Sample size calculation was performed based on the data published by Kaplan et al. [19], who reported that 23.2% out of the patients with severe sepsis and septic shock who had one CVC inserted developed VTE. This study was considered for sample size calculation because it reports the incidence of VTE in patients with both one and two concurrent CVCs. Consequently, the necessary sample size was estimated to be 109 patients, for the following: α = 0.05, p0 = 0.23, p1 = 0.46, power = 0.8.

### 2.2. Data Collection

The following data have been collected for all the patients: demographics (age, gender, smoking status), days of hospitalization in the ICU, anticoagulation or antiaggregating therapy, comorbidities (COVID-19, heart failure, chronic venous insufficiency, chronic obstructive pulmonary disease, chronic kidney disease, neurological disorders, cirrhosis, active cancer, hematologic diseases), laboratory tests (hemoglobin, white blood cells, thrombocytes, C-reactive protein, creatinine, procalcitonin, international normal ratio, fibrinogen), vitals (SpO_2_), arterial blood gases (pH, PaO_2_, lactate), therapeutic interventions (two catheters in the same vein, single catheter, location of the catheters, plasma exchange, CVV-HDF), complications (minor bleedings, central line-associated bloodstream infections (CLABSIs), numerous punctures, catheter malposition). The Caprini risk score was calculated according to the Resources section on the Caprini Risk Score website [20]. The Acute Physiology Score and Chronic Health Evaluation (APACHE) II was determined on the first day of the ICU admission, using MD Calc’s APACHE II Score calculator available online [21].

### 2.3. Data Analysis

Normality of data was tested using the Shapiro–Wilk test. For numerical variables, the report provides the mean and interquartile range (IQR), while categorical variables are presented with counts and percentages. The Chi square statistical test (either asymptotic or Monte Carlo simulation with 10,000 samples) was used to assess the statistical significance of the association between the categorical variables. Either Mann–Whitney U or Kruskal–Wallis statistical tests were employed to assess the distribution of the numerical variables.

Odds Ratios (ORs) were calculated for the outcomes. In a subsequent step, logistic regression models were employed to assess the ongoing relevance of the relationship and explore potential causal connections.

The data analysis was performed using SPSS v26, while the graphical representations and power analysis (for sample size calculation) were performed in R v 4.3.2 (packages “webPower”, “ggplot2” and “forestplot”). All the reported values are two-tailed, and a statistical significance threshold of 0.05 (95% confidence level) was applied.

### 2.4. Ethics

This study was conducted in accordance with the Declaration of Helsinki, and the protocol was approved by the Ethics Committees of the Resita County Emergency Hospital (1424/30.01.19). The collected data were de-identified before conducting the statistical analysis.

## 3. Results

### 3.1. Descriptive Statistics

The final version of the database enclosed 114 patients. Among the numeric variables, only the Caprini risk score had a normal distribution.

A majority of the patients were elderly, males and had COVID-19, as shown in Table 1. Patients either had two CVCs in the same vein or had their catheter changed due to local complications.

The femoral vein was the preferred site for the insertion of the CVCs (62.3% of the cases). Two concurrent CVCs were placed in 52.6% of patients. CVV-HDF was required in 41.7% of the patients included. A great majority of patients (86.8%) received anticoagulants, a measure taken due to the high Caprini score values of the individuals. Patients who did not receive this medication had specific contraindications.

Supplementary Tables S1–S3 provide an overview of the laboratory findings, parameters employed in Caprini risk score assessment, and patients’ comorbidities. In three out of seven patients with two concurrent CVCs and active cancer, CVV-HDF was needed.

**Figure 2:**
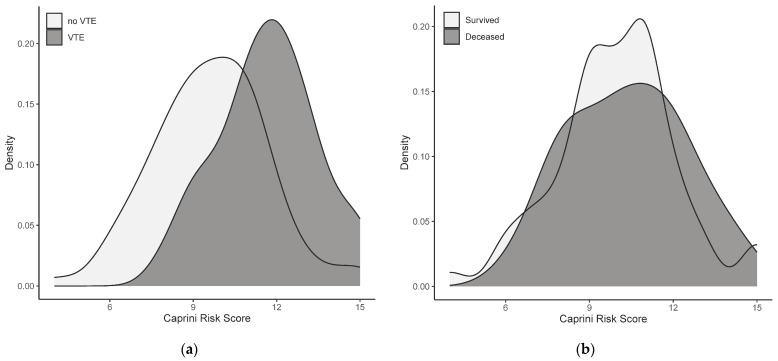
Density plots for the Caprini risk score: (**a**) main outcome; (**b**) secondary outcome.

Density plots for the Caprini risk scores are depicted in Figure 2. The Caprini risk score was higher among patients whose evolution was complicated by VTE (*p* < 0.001). Conversely, patients who deceased did not present higher values of the Caprini risk score (*p* = 0.377) in comparison to those who survived.

Patients who had their CVC replaced had the Caprini risk score ranging between 9 and 11.5 points. The IQRs of the Caprini risk score for each CVC insertion situs were as follows: 9–12 points for the femoral vein, 9–10 for the subclavian vein, 8–11 for the internal jugular vein. The group of patients with two concurrent CVCs had increased values of the score (IQR 9.5 to 12, in comparison to 8 to 11 for the patients with only one CVC, *p* < 0.001). Patients that received both anticoagulant and antiaggregant therapy had significantly higher values of the score (*p* = 0.036). Besides VTE, other catheter insertion complications were bleeding, CLABSIs, multiple punctures and malposition. The high frequency of CLABSIs, especially in patients with two CVCs, is of note even though it does not reach statistical significance (Table 2).

Descriptive statistics for the main outcome (VTE) and the secondary outcome (in-hospital mortality) are shown in Table 3. The highest occurrence of VTE was observed among patients with COVID-19 who underwent the insertion of two CVCs (26.6% vs. 7.4%; *p* = 0.006). A significant difference in the frequency of the VTE was noted while comparing patients with one CVC to those with two CVCs (*p* = 0.041, asymptotic Chi-square statistical test). Furthermore, neither COVID-19 nor two CVCs influence the patient’s outcome when analyzed in isolation.

**Figure 3:**
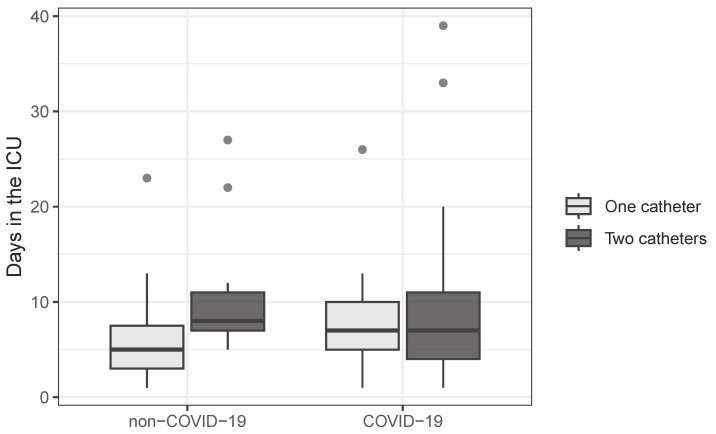
Box plots for the ICU admission length, showing minimal variability. The extreme case in the COVID-19 group is a 70-year-old patient with multiple comorbidities who died.

Box-plots illustrating the length of the hospitalization in the ICU are depicted in Figure 3. The difference between the ICU hospitalization time of the patients with a single CVC and those with two CVCs is statistically significant only in the group of patients without COVID-19 (*p* = 0.005). Patients with COVID-19 had similar lengths of ICU stay regardless of the number of catheters they had inserted.

There was no discernible variance observed in the distribution of APACHE II and qSOFA scores between patients with singular versus those with two concurrent CVCs.

### 3.2. Analysis of the Outcomes

**Figure 4:**
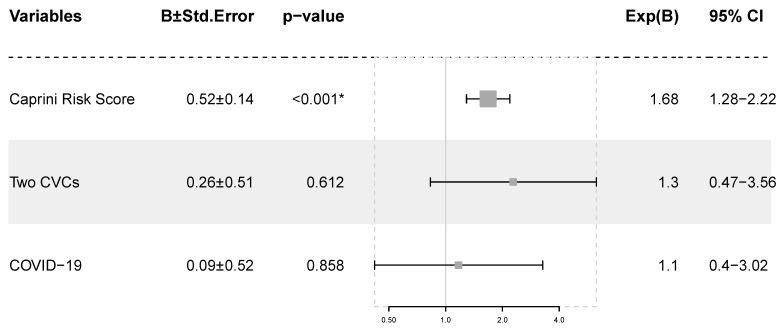
Logistic regression Model 1. The squares correspond to the ORs, while the lines represent the 95% CIs. Abbreviations: B ± Std.Error—regression coefficient ± standard error; CI—confidence interval; CVC—central venous catheter; OR—odds ratio; *—statistical significance, *p* < 0.01.

All the patients who developed VTE during their ICU admission had a Caprini risk score equal to or above 9. More than half of the patients with VTE had both COVID-19 and two central venous catheters in the same vein. The odds of developing thrombosis in the study group were 2.5 times higher in patients with two central venous catheters in the same vein (95% CI: 1.02–6.12; *p* = 0.041). A similar association between COVID-19 and VTE was not proven. In order to test the robustness of these findings, we utilized a logistic regression model (the findings are presented in Figure 4). The Caprini risk score is the only predictor that holds a powerful statistical significance. The Nagelkerke R^2^ is 0.304.

Fourteen patients with two concurrent CVCs were not treated with CVV-HDF (they received plasmapheresis), and two patients that received CVV-HDF did not have two CVCs. Out of the 14 patients mentioned, only one developed thrombosis (*p* = 0.023, Monte Carlo simulation with 10,000 samples). Consequently, we proposed two supplementary regression models. In the first one, having two CVCs placed was the predictor, while sex (male), active cancer, the time spent in the ICU and the APACHE II score were covariates. The second model used the same covariates, but the predictor was CVV-HDF. Table 4 highlights the higher predictive value of Model 2B and the higher risk of developing VTE of the patients that had undergone CVV-HDF.

The risk of in-hospital death was analyzed in a similar way to the main outcome. Various logistic regression models were tested; however, none proved capable of accurately predicting mortality within this patient cohort.

## 4. Discussion

Although the simultaneous placement of two CVCs in the same vein presents advantages in specific clinical scenarios, healthcare providers must also consider potential complications. Therefore, this procedure must be recommended or performed according to the patient’s needs.

The relationship between catheter insertion and thrombosis has been proven by multiple studies. Zhang et al. concluded in a prospective study on 281 patients that CVC use, the Caprini score and ICU length of stay are risk factors for developing VTE in the ICU admitted patients [22]. In another prospective study that included 113 patients with severe sepsis and septic shock, the presence of a CVC increased the risk of developing VTE by 4.37 times (95% CI, 1.77–10.74; *p* = 0.001) [19]. The presence of a CVC was also proven to be the strongest independent predictor for upper extremity deep vein thrombosis (OR, 7.3; 95% CI, 5.8–9.2) [23]. Kuang et al. analyzed 1643 critically ill patients and reported an incidence of symptomatic thrombotic complications of the CVCs of 9.5 per 1000 catheters days [4]. Joks et al. reported an incidence of 13.5% of CVC-related VTE in hematological patients [5], whereas in oncology patients, VTE’s occurrence appears to range around 5% [6]. Spitzer et al. found no significant differences regarding the incidence of CVC compli-cations while comparing 97 patients with two CVCs simultaneously placed in the right internal jugular vein and 63 patients with exclusive dialysis catheter insertion [24].

The importance of accurately assessing a patient’s risk for catheter-related VTE lies in the elevated occurrence of complications, notably, pulmonary embolism and post-thrombotic syndrome. The incidence of pulmonary embolism has been documented to be as high as 17%, with instances where pulmonary embolism resulting from CVC-related thrombosis was the patients’ death cause [25]. Additionally, the same study highlights the incidence of post-thrombotic syndrome to reach as high as 80%.

In our study, all patients experienced septic shock, and each had a Caprini risk score exceeding 9 points, indicating a highly elevated risk of thrombosis. A systemic review conducted by Hayssen et al. emphasized that most authors use a four-risk-category-stratification for VTE, with the highest risk category cutoff varying from ≥5 points to ≥10 points [14]. According to these classifications, 100 patients can be classified as highest risk with a cutoff score of ≥9 points and 88 patients with a cutoff of ≥10 points. The Caprini risk score was 1.2 times higher among patients whose evolution was complicated by VTE (*p*-value < 0.001), similarly to data from the literature [16]. Deceased patients were not associated with increased values of the Caprini risk score, which suggests that mortality in our cohort is independent of the VTE risk.

In a study that included 1030 patients with COVID-19, Chen et al. described a significantly higher mortality in patients with a high Padua, IMPROVE and Caprini VTE score and an increased risk of VTE in patients with a higher sequential organ failure assessment (SOFA score) [16]. In our study, VTE mostly occurred in patients with COVID-19 who had two CVCs. This can be correlated with the documented increased risk of thrombosis in COVID-19 patients, described in other studies [5,26,27,28], along with a higher frequency of VTE in patients with two CVCs compared to those with a single catheter (*p* = 0.041). Neither COVID-19 nor two concurrent CVCs could efficiently predict the final outcome of the patients from our cohort.

When analyzing hospitalization periods, it can be observed that non-COVID patients who undergo the simultaneous placement of two CVCs experience prolonged ICU stay compared to those with singular catheter insertion (*p* = 0.005). COVID-19 patients had similar lengths of ICU admissions regardless of the number of central catheters they had inserted. Given that the length of ICU admission for patients with severe forms of COVID-19 or acute respiratory distress syndrome from other causes was similar in other reports [29], the prolonged hospitalization for non-COVID patients can be attributed either to the severity of their disease, necessitating the placement of a second catheter for hemodialysis or plasmapheresis, or the deterioration of their condition due to thromboembolic complications. Another explanation could be the prolonged requirement for mechanical ventilation in certain isolated cases.

CLABSIs were the most frequent complications (besides VTE) in our study. Their frequency was three times higher in the group of patients with two concurrent CVCs, the difference being marginally significant. Dube et al. concluded in a study of over 50,000 patients that the risk of bloodstream infections in patients with two CVCs was approximately 80% [30]. The elevated frequency of catheter-related infections in our patient cohort can also be ascribed to the prevailing utilization of the femoral vein as the primary site for catheter insertion. This was likely a result of the critical condition, namely, respiratory distress, that the patients presented with upon arrival in the emergency department. The elevated risk of infections associated with central venous catheterization in the femoral vein was also documented in other studies [31,32]. Moreover, an almost tripled risk of developing VTE for patients with CLABSIs has been reported [8].

To test the risk of VTE in patients with pulmonary septic shock, we built a logistic regression model, in which we used two CVCs in the same central vein, SARS-CoV-2 infection and the Caprini score as predictors. The latter seems to be the only statistically significant predictor, the risk of developing VTE increasing by 6.8% for each additional Caprini risk score point. The low statistical significance of having two concurrent CVCs when compared to the Caprini risk score might be caused by the interaction between the two variables (the scoring system accounts for CVV-HDF).

Considering that the Caprini risk score is an already established powerful tool to assess the risk of thrombosis that considers a handful of factors, we went further with the analysis and proposed two more logistic regression models. Our aim was to compare the predictive values of two concurrent CVCs (Model 2A) and CVV-HDF (Model 2B). This was feasible because not all the patients that had two CVCs ended up receiving renal replacement therapy, and not all the patients that were treated using CVV-HDF had two CVCs inserted in the same vein. As we mentioned before, the second CVC served as an access route for CVV-HDF and was placed anticipatively.

The decision to start the renal replacement therapy was taken secondary to the development of acute kidney injury, a pathology with an increased mortality in the ICU [33]. The selection of the most appropriate type of renal replacement therapy follows a personalized approach. Several factors, including patients’ clinical condition, the therapeutic goals and equipment availability, should be used to determine the most suitable technique [34]. CVV-HDF is an adequate procedure for hypotensive patients [35]. Furthermore, considering that active cancer was associated with an increased risk of thrombosis in patients who had CVCs inserted [36,37], it was decided to use active cancer status as a covariable. Supplementary, we also used the male sex and the ICU hospitalization length as covariates. To account for the severity of the patients’ condition, the APACHE II score, recognized as the gold standard prognostic stratification system [38], was incorporated as a covariate. Thus, in model 2A, for patients of the same sex, with a specific oncological condition, the same period of hospitalization and similar disease severity, the insertion of two CVCs increases the risk of VTE by 2.3 times, with a marginally significant *p*-value and a not satisfying 95% CI. At the same time, in model 2B, using CVV-HDF increases the risk of VTE by almost five times, while controlling for the same factors. In addition, the difference between the Nagelkerke R^2^ values of the two models emphasizes the better predictability potential of the model 2B. Summarizing, the increased risk of thrombosis in our study could be secondary to the insertion of the hemodialysis catheter and not necessarily to the insertion in the same central vein. An explanation for the increased risk of thrombosis associated with CV-HDC might be the turbulent flow beyond the catheter tip, resulting from increased flow compared to the infusion catheter. This turbulence triggers local endothelial proliferation, fostering fibrosis lesions, stenosis, and subsequent thrombosis [3].

The incidence of central vein stenosis associated with CV-HDC has been reported to range between 20% and 40%, with the higher risk being directly proportional to the duration of catheter retention and its placement in the left internal jugular or subclavian vein compared to the right internal jugular or femoral vein [3].

The analysis of in-hospital mortality followed a similar approach as the primary outcome. As the incidence of deep vein thrombosis rose, a corresponding increase in the duration of mechanical ventilation, hospitalization, and overall mortality was reported [39]. Several logistic regression models were examined, but none demonstrated the ability to reliably predict mortality within this patient cohort. This was attributed to the fact that the increased risk of death was not directly linked to central venous catheterization. Instead, it could be associated with the acute pathology of the patient, along with the comorbidities that intensified its severity and necessitated the placement of one or two CVCs in the same vein.

Our study has certain limitations. Firstly, its retrospective design relied on data collected from medical records registered in the SARS-CoV-2 pandemic. During that time span, ICU wards were overcrowded, and staffing was limited. Therefore, some data from the medical records may be lacking. Secondly, some patients may not have undergone contrast chest-CT for confirming the pulmonary embolism due to their critical condition. As a result, we examined both suspected and confirmed cases to mitigate this risk. Thirdly, given that the patients receiving CVCs were often in critical condition and managed in the ICU, it is challenging to definitively attribute the outcomes of VTE to either the device or the severity of the patient’s pathology. Lastly, the limited sample size of 114 patients was another constraint of this study, given the intention to exclusively include patients with pulmonary septic shock. Furthermore, with a mean age of 73 years and the presence of pulmonary septic shock leading to an elevated Caprini risk score, the assessment of the effectiveness of the Caprini risk score for VTE risk in patients with more than one central line inserted during ICU stay was limited.

Despite these limitations, particularly the small sample size, the findings from this study could hold significance for ICU physicians. They should not refrain from inserting two catheters into the same central vein due to catheter-related complications for critically ill patients with septic shock who spend prolonged ICU admission and often require multiple punctures for CVC placement/exchange, who clearly need both perfusion and dialysis catheters from the beginning. The risks persist regardless of whether they are positioned in separate central veins. Instead, they should closely monitor the patient’s condition, remaining vigilant in the prevention and early treatment of VTE. Members and consultants of the American Society of Anesthesiologists agree that the decision ultimately lies with the case physician and should be customized based on the patient’s condition [2].

## 5. Conclusions

The insertion of two concurrent CVCs is rare but useful in the ICU for patients with septic shock and invariably comes with some risks. The risk of developing VTE while having two CVCs in the same vein is increased. However, this increase seems to be caused by the nature of the second catheter (used for CVV-HDF) rather than its mere presence. Neither the mortality nor the ICU hospitalization period were increased by the presence of two CVCs.

## Figures and Tables

**Table 1:** Descriptive statistics.

Variable		All Patients (*n* = 114)
Age ^(a)^		73.5 (63–82)
Sex		
	Female ^(b)^	42 (36.8%)
	Male ^(b)^	72 (63.2%)
Active smoker ^(b)^		5 (4.4%)
Caprini risk score ^(a)^		10 (9–12)
COVID-19 ^(b)^		76 (66.7%)
Active cancer ^(b)^		11 (9.6%)
Two concurrent CVCs ^(b)^		60 (52.6%)
CVC location		
	Femoral vein ^(b)^	71 (62.3%)
	Subclavian vein ^(b)^	13 (11.4%)
	Internal jugular vein ^(b)^	50 (43.9%)
SpO2 [%] ^(a)^		80.5 (74–87)
PaO2 [mmHg] ^(a)^		43.95 (32.8–69.4)
Lactate [mmol/L] ^(a)^		2.25 (1.53–3.4)
White blood cells [10^3^/μL] ^(a)^		15.65 (9.99–18.98)
C reactive protein [ng/mL] ^(a)^		88.03 (25.09–149.65)
Plasma Exchange ^(b)^		32 (27.8%)
CVV-HDF ^(b)^		48 (41.7%)
Antiplatelet treatment ^(b)^		66 (57.9%)
Anticoagulation ^(b)^		99 (86.8%)
APACHE II score ^(a)^		27 (25–29)
qSOFA score		
	1 point ^(b)^	9 (7.9%)
	2 points ^(b)^	80 (70.2%)
	3 points ^(b)^	25 (21.9%)

^(a)^—median (IQR); ^(b)^—counts (percentages). Abbreviations: APACHE, acute physiology score and chronic health evaluation; CVC—central venous catheter; CVV-HDF—continuous venovenous hemodiafiltration; IQR—interquartile range; PaO_2_, partial pressure of oxygen in arterial blood; SpO_2,_ peripheral oxygen saturation on room air at hospital admittance; qSOFA—quick sequential organ failure assessment.

**Table 2:** Descriptive statistics for complications.

Variable	All Patients (*n* = 114)	One CVC (*n* = 54)	Two CVCs (*n* = 60)	*p*-Value
Minor bleeding	12 (10.5%)	7 (13%)	5 (8.3%)	0.421
CLABSIs	15 (13.2%)	4 (7.4%)	11 (18.3%)	0.073 ^(a)^
Numerous punctures	7 (6.1%)	4 (7.4%)	3 (5%)	0.441
Malposition	7 (6.1%)	2 (3.7%)	5 (8.3%)	0.265

Counts (percentages), either asymptotic Chi-Square statistical test or Monte Carlo simulation with 10.000 samples; ^(a)^—marginally significant, *p* < 0.1. Abbreviations: CVC—central venous catheter.

**Table 3:** Descriptive statistics for the outcomes.

Variable		All Patients (N = 114)	One CVC (N = 54)	Two CVCs (N = 60)	*p*-Value
VTE	All patients	29 (25.4%)	9 (16.6%)	20 (33.3%)	0.041 *
	+COVID-19	20 (26.3%)	4 (7.4%)	16 (26.6%)	0.006 **
Deceased	All patients	50 (43.9%)	19 (35.2%)	31 (51.7%)	0.077
	+COVID-19	36 (47.4%)	11 (30.6%)	25 (69.4%)	0.01 *

Counts (percentages), asymptotic Chi-Square statistical test; *—statistically significant, *p* < 0.05, **—statistically significant, *p* < 0.01. Abbreviations: CVC—central venous catheter; VTE—venous thromboembolism.

**Table 4:** Logistic regression models 2A and 2B.

Variable	B ± Std.Error	*p*-Value	OR	95% CI
Model 2A: Thrombosis~Two CVCs (covariates: ICU Days, active cancer, male sex, APACHE II score) Nagelkerke R^2^ = 0.102				
Two CVCs	0.86 ± 0.47	0.068	2.36	0.94–5.91
Model 2B: Thrombosis~CVV-HDF (covariates: ICU Days, active cancer, male sex, APACHE II score) Nagelkerke R^2^ = 0.193				
CVV-HDF	1.55 ± 0.48	0.001	4.73	1.84–12.2

Abbreviations: B ± Std.Error—regression coefficient ± standard error; CI—confidence interval; CVC—central venous catheter; CVV-HDF—continuous veno-venous hemodiafiltration; ICU—intensive care unit; OR—odds ratio.

## Data Availability

The data sets are not publicly available, but de-identified data may be provided upon request from Cosmin Iosif Trebuian.

## Supplementary Materials

The following supporting information can be downloaded at: https://www.mdpi.com/article/10.3390/medicina60050785/s1, Table S1: Laboratory findings; Table S2: The Caprini Risk Score; Table S3: Comorbidities.

## Abbreviations

APACHE	acute physiology and chronic health evaluation
CI	confidence interval
CLABSI	central line-associated bloodstream infections
COVID-19	coronavirus disease-19
CT	computed tomography
CVC	central venous catheters
CV-HDC	central venous hemodialysis catheters
CVV-HDF	continuous venovenous hemodiafiltration
ICU	intensive care unit
IQR	interquartile range
MAP	mean arterial pressure
OR	odds ratio
PaO_2_	partial pressure of oxygen in arterial blood
PCT	procalcitonin
SARS-CoV-2	severe acute respiratory syndrome-coronavirus-2
SpO_2_	peripheral oxygen saturation on room air at hospital admittance
Std	standard
VTE	venous thromboembolism
qSOFA	quick sequential organ failure assessment
