# Supplementary material for: Risk Assessment of Venous Thromboembolism among Septic Shock Patients: Single versus Concurrent Insertion of Central Venous Catheters
Source: Medicina (Kaunas). 2024 May 9;60(5):785. doi: 10.3390/medicina60050785 (PMC11123159; doi:10.3390/medicina60050785)
Supplement: Supplementary file 1 [file medicina-60-00785-s001.zip › medicina-2966513-supplementary.pdf]

Table S1: Laboratory findings

Variable	All patients (N = 114)	One CVC (N = 54)	Two CVCs (N = 60)
Creatinine [mg/dL]	1.13 (0.78 – 1.86)	1.14 (0.87 – 2.12)	1.13 (0.76 – 1.72)
Fibrinogen [mg/dL]	407 (279.5 – 494.5)	358 (256 – 490)	413 (284 – 499)
Hemoglobin [g/dL]	13.3 (11.35 – 14.35)	13.3 (11.5 – 14.2)	13.15 (11.2 – 14.4)
INR	1.31 (1.13 – 1.42)	1.26 (1.02 – 1.4)	1.31 (1.16 – 1.42)
pH	7.43 (7.36 – 7.47)	7.36 (7.3 – 7.45)	7.44 (7.37 – 7.49)
Procalcitonin [ng/mL]	0.74 (0.69 – 0.82)	0.74 (0.7 – 0.78)	0.74 (0.68 – 0.94)
Thrombocytes [10^3/μL]	245 (161 – 361)	227 (143 – 316)	268 (179 – 369)
WBC [10^3/μL]	15.65 (9.99 – 19.84)	12.93 (9.05 – 18.23)	16.27 (11.41 – 21.58)

Median (IQR), WBC, White blood cells.

Table S2: The Caprini Risk Score

Points	Variable	All patients (N = 114)	One CVC (N = 54)	Two CVCs (N = 60)
1	41-60 years old	15	10	5
	Bed rest <3 days	46	28	18
	COPD	21	11	10
	Diabetes	49	23	26
	Heart failure	34	17	17
	Obesity	41	28	13
	Septic shock	114	54	60
	Smoking	37	21	16
2	>3 days bed rest	66	24	42
	Age 61-74	62	26	36
	CVC insertion	114	54	60
	Hematological disease	37	20	17
	Malignancy	11	4	7
3	Above 75 years old	32	13	19
	Chronic venous insufficiency	7	2	5

Counts

Table S3: Comorbidities

**Variable**	**All patients** (N = 114)	**One CVC** (N = 54)	**Two CVCs** (N = 60)
Diabetes mellitus	49 (43%)	23 (42.6%)	26 (43.3%)
Hematologic diseases	39 (34.2%)	17 (31.5%)	22 (36.7%)
Heart failure	34 (29.8%)	17 (31.5%)	17 (28.3%)
Neurological disorders	32 (28.1%)	4 (7.4%)	28 (46.7%)
Cirrhosis	32 (28.1%)	13 (24.1%)	19 (31.7%)
COPD	22 (19.3%)	12 (22.2%)	10 (16.7%)
Chronic kidney disease	15 (13.2%)	6 (11.1%)	9 (15%)
Active cancer	11 (9.6%)	4 (7.4%)	7 (11.7%)
Chronic venous insufficiency	7 (63.2%)	2 (3.7%)	5 (8.3%)

Counts (percentages)
